# The impact of the COVID-19 pandemic on pediatric developmental services: a cross-sectional study on overall burden and mental health status

**DOI:** 10.1186/s13690-022-00876-5

**Published:** 2022-04-08

**Authors:** Peter Borusiak, Yuliya Mazheika, Susanne Bauer, Edda Haberlandt, Ilona Krois, Christian Fricke, Liane Simon, Petra Beschoner, Lucia Jerg-Bretzke, Franziska Geiser, Nina Hiebel, Kerstin Weidner, Christian Albus, Eva Morawa, Yesim Erim

**Affiliations:** 1 Witten/Herdecke University, Witten, Germany; 2 Wagener-Foundation for Social Pediatrics, Emsdetten, Germany; 3 Aks - Arbeitskreis für Vorsorge- und Sozialmedizin, Bregenz, Austria; 4 Kinderklinik Dornbirn, Dornbirn, Austria; 5 Helios Hospital Krefeld, Krefeld, Germany; 6 VIFF – Vereinigung für Interdisziplinäre Frühförderung – Bundesvereinigung, Hamburg, Germany; 7 Hochschule Nordhausen - University of Applied Sciences (UAS), Nordhausen, Germany; 8 MSH Medical School Hamburg, Hamburg, Germany; 9 Department of Psychosomatic Medicine and Psychotherapy, Ulm University Medical Center, Ulm, Germany; 10 Department of Psychosomatic Medicine and Psychotherapy, University Clinic of Bonn, Bonn, Germany; 11 Department of Psychotherapy and Psychosomatic Medicine, Faculty of Medicine, Technische Universität Dresden, Dresden, Germany; 12 Department of Psychosomatics and Psychotherapy, University Hospital of Cologne, Cologne, Germany; 13 Department of Psychosomatic Medicine and Psychotherapy, University Hospital of Erlangen, Friedrich-Alexander University Erlangen-Nürnberg (FAU), Erlangen, Germany

**Keywords:** Developmental pediatrics, Psychological strain, Anxiety, Depression, COVID-19, Mental health

## Abstract

**Background:**

The COVID-19 outbreak has taken a heavy toll on the mental well-being of healthcare workers, even those who have not been directly involved in the care of acutely ill patients. The aims of this study were to identify the overall burden and mental health status of healthcare workers in pediatric developmental services under the influence of the COVID-19 pandemic, and to identify the risk and protective factors associated with mental health.

**Methods:**

This cross-sectional web-based study was part of a large multicenter VOICE study conducted among employees ((neuro-)pediatricians, psychologists, speech therapists, occupational therapists, etc.) from various pediatric developmental services between June and July 2020. A total of 1291 questionnaires regarding overall burden, mental health status (depression, generalized anxiety disorder and emotional exhaustion) and risk and protective factors for mental health (working conditions, potential problems during the COVID-19 pandemic and psychological resources) were analyzed. Descriptive statistics and multiple linear regression were used for data analysis.

**Results:**

A total of 44.5% (574/1291) participants felt a high or very high overall burden during the COVID-19 pandemic. Of all the participants, 14.6% (171/1173) reported clinically significant levels of depressive symptoms, 17.0% (199/1173) reported generalized anxiety disorder symptoms and 44.6% (532/1192) reported emotional exhaustion. Multiple linear regression analyses identified several common risk and protective factors for mental health status variables. The burden of an increase in the quantity of work, fear of work and fear of becoming infected showed the strongest negative associations, whereas psychological resources and sufficient relaxation in leisure time exhibited the strongest positive associations.

**Conclusion:**

Employees who were not directly involved in the care of acutely ill patients were also exposed to considerable stress, some of which was not different from that experienced by professionals who were directly affected. These employees should not be lost sight of and must be offered appropriate support.

## Background

**Fig. 1:**
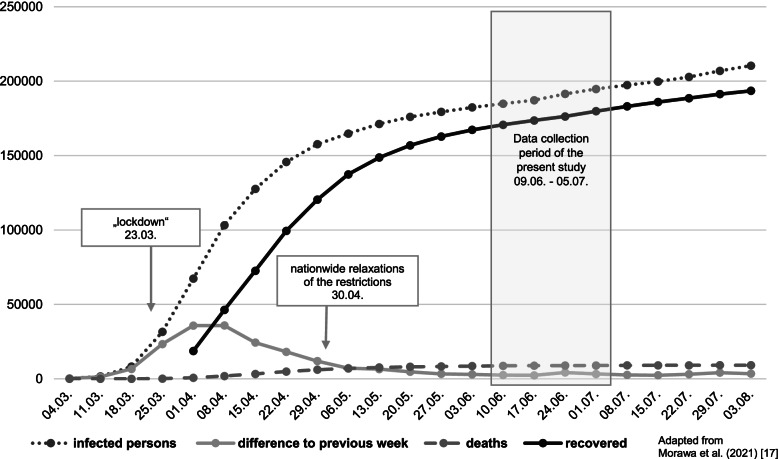
Timing of data collection in our study in the context of the COVID-19 pandemic situation in Germany

The COVID-19 pandemic has affected nearly everyone’s life. Although, fortunately, only a minority was directly affected by the virus (Fig. 1), most people were involved directly or indirectly by the restrictions that were introduced due to the pandemic. The psychological burden and well-being of healthcare workers (HCWs) have been addressed in several publications, which have consistently revealed a high level of stress, anxiety, and depressive symptoms [1]. Different, and sometimes contradictory, individual stress factors were identified in these studies.

There is an increasing number of reports covering psychological effects as well as mental health symptoms during different stages of the pandemic. Many studies were carried out during peak incidences of COVID-19 infections or immediately during lockdown phases, focusing either on the whole population with the participation of volunteers, on different groups of patients with distinctive diseases or on HCWs who were mostly directly confronted with (possibly) infected patients [2–4]. Most, if not all, of the HCWs demonstrated elevated rates of clinically significant symptoms of anxiety, depression, insomnia, and a high level of emotional exhaustion as well as an increased abuse of alcohol and drugs [5, 6]. A recent review found 51 studies that investigated the impact of the pandemic on healthcare workers [7]. The vast majority of these studies were from China or low- and middle-income countries (LMICs), with the number of people studied ranging from 52 to 14,825 and a median sample size of 548. Most of these studies found significantly elevated levels of anxiety, depression, burnout and distress. Even if there is evidence for certain risk groups or profiles, the data are still not always entirely consistent. In addition, other studies have reported an increase in the burnout symptom of emotional exhaustion [6, 8]. Burnout has a very strong impact on both the well-being and health of workers, and on the efficiency of their organization and their work [9]. Usually, burnout occurs in employees who are exposed to long-term occupational risk factors. However, it has also been demonstrated that stressful emergences, such as pandemics, can also easily trigger emotional exhaustion [10].

As in most countries, the whole efforts of the healthcare system in Germany were directed towards coping with real or potential shortages of acute medical supplies. Collateral damages were reported in different settings, diseases and conditions stretching from, for example, cardiac arrest [11] and immunizations [12] to the interruption of therapeutical services, such as physiotherapy or psychotherapy. Many publications have focused on nurses and physicians treating patients with COVID-19 or other acute diseases. Of course, those “frontline providers” deserve special attention [13]. Yet, we also want to offer a closer look into HCWs in the “second line” caring for children and adolescents with chronic somatic, mental and developmental conditions.

In Germany, a special health care system exists for children with complex chronic conditions or developmental disorders. There are approximately 160 Social Pediatric Centers (SPCs) that provide care with an interdisciplinary approach and multidisciplinary team consisting of pediatric neurologists, psychologists, speech therapists, occupational therapists, physiotherapists, social workers, music therapists and remedial teachers [14]. Other hallmarks of SPCs include a smaller emphasis on somatic aspects and a high proportion of psychological and psychosocial interventions using a family integrated/centered approach, and the availability of continuous long-term treatment until adulthood with networking (e.g., educational services, public health services, etc.). For preschool-aged children with developmental problems, there exist approximately 1000 early intervention centers (EICs) for developmental health promotion [15]. Some EICs provide a remedial holistic approach, while others offer additional medical therapeutic interventions, such as speech therapy, occupational therapy and physiotherapy, as a composite service. In addition to the two areas of work already mentioned, we also included HCWs from the pediatric section of aks (Arbeitskreis für Vorsorge- und Sozialmedizin/the working group for preventive and social medicine). The latter more or less represents a service that adequately combines both German institutions for the region of Vorarlberg (Austria), which covers roughly 400,000 inhabitants (among them approximately 80,000 children and adolescents under 18 years old) [16].

During the first shutdown in Germany, most institutions were subject to restrictions. The COVID-19 outbreak also forced many pediatric developmental services to adjust or completely stop their work during the first weeks of the pandemic. As a result, employees also confronted numerous changes in the workplace (e.g., working from home, switching to alternating shift work and/or transferring to another ward in the hospital), which could also be associated with uncertainties and fears. To the best of our knowledge, there have been no studies to date that have examined the impact of the COVID-19 pandemic on staff working in interdisciplinary teams with chronic pediatric patients. In our study, we aimed to fill this gap by examining the overall burden and mental health status of employees caring for children with complex chronic conditions or developmental disabilities during the COVID-19 pandemic. Specifically, we aimed to address the following questions:

- How did employees’ self-perceived overall burden levels change before (retrospectively) and during the COVID-19 pandemic?
- What is the prevalence of clinically significant levels of depressive symptoms, generalized anxiety disorder symptoms and emotional exhaustion among employees?
- Which risk and protective factors are associated with depressive symptoms, generalized anxiety disorder symptoms and emotional exhaustion?

## Methods

This study was part of a large multicenter study with a network of researchers from different clinical settings and five university hospitals who started the “VOICE study” on the mental health of medical personnel during the COVID-19 pandemic situation; the study is registered in the German Register for Clinical Studies (DRKS00021268). The VOICE study is embedded in the egePan Unimed study as a part of the Network University Medicine (NUM), and is funded by the Federal Ministry of Education and Research (BMBF, funding number: 01KX2021).

### Data collection and participants

The complete VOICE online survey was conducted with different HCWs between April 20 and July 5 2020 by the psychosomatic departments of the university hospitals of Erlangen, Bonn, Ulm, Cologne and Dresden, and was shared via mailing lists or online platforms. The complete methodology was described previously [17]. We added and modified some of the original items on the survey for the specific conditions of the involved institutions. These items pertained to working environment (SPC, EIC, aks or other) as well as a further differentiation of specific health professions (physicians, psychologists, physiotherapists, occupational therapists, speech therapists, remedial teachers, social education, music therapists, social workers, administrative staff or other). Participants were mainly recruited using different mailing lists from June 9 2020 to July 52,020. Two mailing lists exist for SPCs: one comprising the head physicians of all German SPCs and the other more than 1500 staff members. The head physicians were asked to send the invitation to their employees. For EICs, there is a mailing list that reaches 100 EICs; additionally, the board members of the Federal Association of EICs (Germany has 16 different federal states) were asked to share the invitation. The aks sent out an email to the addresses of approximately 140 employees. The data collection period must be viewed in the context of the development of the COVID-19 pandemic in Germany in terms of the total number of infected, dead and recovered persons and with respect to the difference in the infection caseload in comparison to the previous week. In this study, data collection occurred shortly after the end of the first wave, when the nationwide relaxation of restrictions was possible (Fig. 1). The 15-min survey (77 items) used in our study was programmed with two academic online survey tools: Unipark and SoSci Survey. The inclusion criteria for this part of the study were a minimum age of 18 years, working in the social pediatric care sector, residence/workplace in Germany or Vorarlberg (Austria) and sufficient German language skills. Electronic informed consent was obtained before the survey began. Consent was given by actively ticking the consent checkbox. In the consent form, participants were informed about the aims of the study, voluntariness, anonymity and the handling of the collected data. Participation was voluntary and anonymous, and participants could withdraw from the study at any time. The participants did not have to fear any negative consequences for the refusal or discontinuation of data collection. Since the data were collected anonymously through an external online survey tool, supervisors did not have the opportunity to verify employee participation.

### Instruments

#### Sociodemographic and occupational variables

The online questionnaire contained sociodemographic information and occupational characteristics (Table 1).

**Table 1:** Sociodemographic findings and occupational characteristics of the participants

	Participants (n) ***N*** = 1291	Participants (%) 100%
**Sex**		
Male	121	9.4
Female	1162	90.0
Diverse	2	0.2
Missing values	6	0.5
**Age groups**		
18–30	136	10.5
31–40	250	19.4
41–50	385	29.8
51–60	425	32.9
> 61	89	6.9
Missing values	6	0.5
**Housing situation/ living alone**		
Yes	218	16.9
No	1067	82.6
Missing values	6	0.5
**Caring for chronically ill or handicapped family**		
Yes, in the household	38	2.9
Yes, but not in the household	226	17.5
No	1021	79.1
Missing values	6	0.5
**Children**		
Yes, in the household	637	49.3
Yes, but not in the household	249	19.3
Of whom as a single parent	126	14.2^a^
No	398	30.8
Missing values	7	0.5
**Immigrant background**		
No	1163	90.0
Yes	121	9.4
Missing values	7	0.5
**Risk group (*multiple responses allowed*)**		
Yes, due to age	177	13.7
Yes, due to underlying health condition	227	17.6
No	914	70.8
Missing values	25	1.9
**Working context**		
Social Pediatric Center	509	39.4
Early intervention center	587	45.5
Aks	56	4.3
Other^b^	127	9.8
Missing values	12	0.9
**Profession**		
Physicians	135	10.5
Psychologist	187	14.5
Occupational therapist	99	7.7
Physiotherapist	69	5.3
Speech therapist	201	15.6
Social education	148	11.5
Remedial teacher	199	15.4
Social worker	13	1.0
Administration	54	4.2
Other	170	13.2
Missing values	16	1.2
**Mode of employment**		
Full-time (100%)	494	38.3
Part-time (less than 100%)	781	60.5
Missing values	16	1.2

^a^Percentage with respect to those answering “yes” to the question of having children

^b^“other” includes participants from private practices or participants with multiple working contexts, e.g., working part-time in a SPC and part-time in an EIC

Percentages may not sum to 100 due to rounding

#### Overall burden variables

To determine participants’ self-perceived overall burden before and during the COVID-19 pandemic, two self-constructed items were included: “How burdened did you feel prior the COVID-19 pandemic?” and “How burdened did you feel in the last two weeks including today?” All statements were rated on a 5-point Likert scale (0 = very low to 4 = very high).

#### Mental health status variables

To assess mental health status, validated measures were used in the survey, namely the *Patient Health Questionnaire-2 (PHQ-2), Generalized Anxiety Disorder-2 (GAD-2),* and *Maslach Burnout Inventory-Emotional Exhaustion subscale (MBI-EE).*

The validated German version of the PHQ-4 is an ultra-short form of the PHQ-D that includes four items answered in a 4-point (0 = never to 3 = nearly every day) format: two items measure depressive symptoms (PHQ-2) and two items measure generalized anxiety disorder symptoms (GAD-2). In this study, we analyzed the subscales (PHQ-2 and GAD-2) separately. A sum score of ≥3 for the PHQ-2 and GAD-2 components may indicate clinically significant levels of depressive or anxiety symptoms [18]. The Cronbach’s alpha scores were 0.73 for the PHQ-2 and 0.78 for the GAD-2.

The MBI-EE subscale (9 items) was chosen to assess job stress and emotional exhaustion using the German version introduced by Büssing and Perrar (MBI-D) [19, 20]. Each item was rated on a 7-point Likert Scale (0 = never to 6 = every day). Maslach-recommended cutoffs for the MBI-HSS (Maslach Burnout Inventory-Human Services Survey) with standard cutoffs (low (0–16), moderate (17–26) and high (≥27)) were used to estimate emotional exhaustion [21]. In the present sample, Cronbach’s alpha was 0.91.

#### Risk and protective factor indicator variables

##### Psychological resources

To assess psychological resources, validated measures were used in the survey, including the *ENRICHD Social Support Inventory-Deutsch (ESSI-D), Sense of Coherence Scale (ultra-short form (SOC-3)), Quality of Life (WHOQOL-BREF) and Scale Optimism-Pessimism-2 (SOP-2).*

The ESSI is an instrument for the assessment of perceived emotional social support. We used a German adaptation of the English ESSI with 5 items on a 5-point Likert scale (1 = never to 5 = always). According to the original version, a values sum score ≤ 18 is considered as low social support [22]. In the present sample, the ESSI-D obtained a Cronbach’s alpha of 0.89.

Sense of coherence (SOC) is a psychological resource that helps people adapt to difficult circumstances. In the present study, SOC was assessed using a validated German ultra-short version of the scale (SOC-3). The 3-item model could be answered on a 7-point Likert scale (1–7). Higher sum values indicated a higher feeling of coherence [23]. In the present sample, Cronbach’s alpha was 0.71.

The complete WHOQOL-BREF is a self-assessment instrument consisting of four domains (physical health, mental health, social relations and environment) and a global self-assessment. In our study, we included one question on quality of life: “How would you rate your quality of life today?” Answers could be given on a 5-point Likert scale (1 = very bad to 5 = very good) [24].

Optimism was measured with the German version of the Scale Optimism-Pessimism-2 (SOP-2). It includes one item answered in a 7-point (1 = not at all optimistic to 7 = very optimistic) format [25].

##### Working conditions and potential problems during the COVID-19 pandemic

Additionally, questions concerning working conditions (“sufficient amount of protective clothing including face masks”, “enough staff for the current workload”, “sufficient recreation during leisure time” and “reliability on teammates if it is getting difficult at work”) and potential problems during the COVID-19 pandemic (“fear of becoming infected”, “fear of infecting family”, “fear of work”, “burden of change of work tasks”, “burden of an increase in the quantity of work”, “feeling of being protected by employer”, “increased cigarette consumption” and “increased alcohol consumption”) as potential influencing factors were included. These questions were assessed using a 5-point Likert scale (0 = complete disagreement to 4 = complete agreement).

### Statistical analysis

Data analyses were conducted with the SPSS Statistics 25 software. Descriptive statistics were performed for the participant characteristics (sociodemographic and occupational characteristics), overall burden, working conditions and potential problems during the COVID-19 pandemic, and scores from the psychometric tools.

To identify the protective and risk factors associated with depressive symptoms (PHQ-2), generalized anxiety disorder symptoms (GAD-2) and emotional exhaustion (MBI-EE), multiple linear regression analyses were conducted. We included the following independent variables in the analysis: sociodemographic and occupational characteristics, psychological resources and working conditions and potential problems during the COVID-19 pandemic. Independent variables with a β score ≥ .100 were considered as clinically relevant predictors following classification for correlation coefficients [26]. The significance level was set to *p* < .05 (two-tailed).

## Results

### Participants

A total of 1291 participants from pediatric developmental services completed the survey and were included in further analyses. Table 1 contains the characteristics of the sociodemographic findings and the different occupational characteristics of the participants.

### Overall burden

**Fig. 2:**
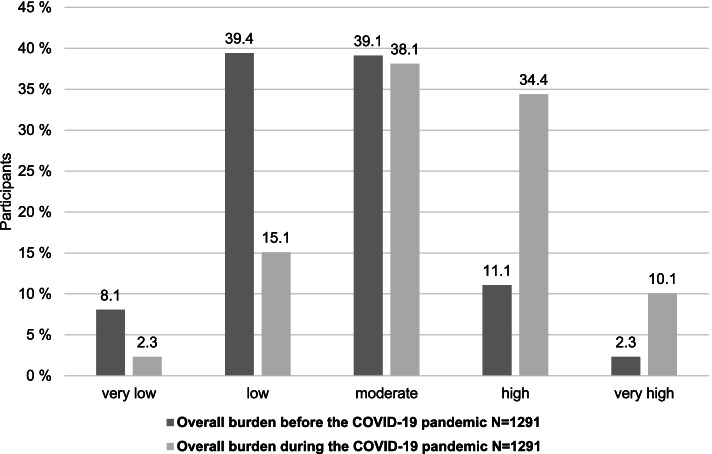
Overall burden associated with COVID-19

Of all the participants (*N* = 1291), 44.5% felt a high or very high overall burden during this phase of the COVID-19 pandemic. The shift toward significantly higher overall burden levels was even more evident when the groups with very low or low overall burden levels were contrasted with those that had high or very high overall burden levels. In this comparison, a significant increase in perceived general high or very high overall burden from 13.4% (173/1291) before the pandemic to 44.5% (574/1291) during the pandemic and a decrease in low or very low overall burden from 47.5% (613/1291) before the pandemic to 17.4% (225/1291) during the pandemic was observed (Fig. 2).

Table 2 provides a closer look at the different professions and potential risk groups, comparing the groups with very low or low overall burden, moderate overall burden and high or very high overall burden.

**Table 2:** Overall burden with respect to profession and risk groups

	Overall burden before the COVID-19 pandemic			Overall burden during the COVID-19 pandemic		
	very low or low	moderate	high or very high	very low or low	moderate	high or very high
	n (%)	n (%)	n (%)	n (%)	n (%)	n (%)
**Profession**						
Physician	52 (38.5)	62 (45.9)	21 (15.6)	30 (22.2)	51 (37.8)	54 (40.0)
Psychologist	97 (51.9)	69 (36.9)	21 (11.2)	47 (25.1)	84 (44.9)	56 (29.9)
Occupational therapist	45 (45.5)	39 (39.4)	15 (15.2)	13 (13.1)	36 (36.4)	50 (50.5)
Physiotherapist	41 (59.4)	23 (33.3)	5 (7.2)	11 (15.9)	27 (39.1)	31 (44.9)
Speech therapist	116 (57.7)	63 (31.3)	22 (10.9)	25 (12.4)	79 (39.3)	97 (48.3)
Social education	50 (33.8)	79 (53.4)	19 (12.8)	26 (17.6)	49 (33.1)	73 (49.3)
Remedial teacher	94 (47.2)	80 (40.2)	25 (12.6)	30 (15.1)	75 (37.7)	94 (47.2)
Social worker	6 (46.2)	4 (30.8)	3 (23.1)	1 (7.7)	5 (38.5)	7 (53.8)
Administration	30 (55.6)	18 (33.3)	6 (11.1)	10 (18.5)	14 (25.9)	30 (55.6)
Other	75 (44.1)	62 (36.5)	33 (19.4)	31 (18.2)	63 (37.1)	76 (44.7)
**Total**	**606 (47.5)**	**499 (39.1)**	**170 (13.3)**	**224 (17.6)**	**483 (37.9)**	**568 (44.5)**
**Risk group** *(****multiple responses allowed)***						
Yes, due to age	73 (41.2)	79 (44.6)	25 (14.1)	21 (11.9)	61 (34.5)	95 (53.7)
Yes, due to underlying health condition	97 (42.7)	88 (38.8)	42 (18.5)	23 (10.1)	72 (31.7)	132 (58.1)
No	452 (49.5)	349 (38.2)	113 (12.4)	181 (19.8)	366 (40.0)	367 (40.2)
**Total**	**622 (47.2)**	**516 (39.2)**	**180 (13.7)**	**225 (17.1)**	**499 (37.9)**	**594 (45.1)**

Percentages may not sum to 100 due to rounding

Compared to the medical-therapeutic fields (occupational therapists, physiotherapists, etc.) and administrative staff, psychologists and physicians showed a smaller increase in overall burden. Those who assigned themselves to a risk group showed higher levels of overall burden in the context of the pandemic.

### Mental health status

#### Prevalence of clinically significant levels of depressive symptoms, generalized anxiety disorder symptoms and emotional exhaustion

The prevalence of clinically significant levels of depressive symptoms, generalized anxiety disorder symptoms and emotional exhaustion are presented in Table 3.

**Table 3:** Levels of depressive symptoms, generalized anxiety disorder symptoms and emotional exhaustion

Scale	n	%
**Depressive symptoms**		
**PHQ-2**^**a**^**:** Sum score range (0–6)	Mean (SD) 1.41 (±1.29)	
Scale values < 3	1002	85.4
Scale values ≥3	171	14.6
**Generalized anxiety disorder symptoms**		
**GAD-2**^**a**^**:** Sum score range (0–6)	Mean (SD) 1.48 (±1.39)	
Scale values < 3	974	83.0
Scale values ≥3	199	17.0
**Emotional exhaustion**		
**MBI-EE**^**b**^**:** Sum score range (0–53)	Mean (SD) 17.07 (±10.83)	
0–16 (low)	660	55.4
17–26 (moderat)	291	24.4
27 or over (high)	241	20.2

^a^[18]

^b^[21]

Valid / missing values for depression symptoms (PHQ-2): *n* = 1173 (90.9%) / *n* = 118 (9.1%)

Valid / missing values for generalized anxiety disorder symptoms (GAD-2): *n* = 1173 (90.9%) / *n* = 118 (9.1%)

Valid / missing values for emotional exhaustion (MBI-EE): *n* = 1192 (92.3%) / *n* = 99 (7.7%)

Abbreviations: *PHQ-2* Patient Health Questionnaire-2, *GAD-2* Generalized Anxiety Disorder, *MBI-EE* Maslach Burnout Inventory-Emotional Exhaustion subscale

*SD* standard deviation

#### Risk and protective factors for depressive symptoms, generalized anxiety disorder symptoms and emotional exhaustion

The mean score in the ESSI-D scale as a measure of perceived social support was 21.3 (±3.5, range 5–25, *n* = 1170). Of those who responded (1170), 959 were above the cutoff, with sum scores > 18. Regarding the basic optimistic attitude as measured by SOP-2 optimism scale, the mean score was 5.3 (±1.2, range 1–7, *n* = 1162). Of all the participants (1162), 884 (76.1%) rated their quality of life as “good” or “very good” at the time of the survey (WHOQOL-BREF, mean score 3.9 ± 0.7, range 1–5, *n* = 1162). The mean value for the SOC-3 was 12.0 (±2.6, range 4–19, *n* = 1167).

A high level of agreement was observed in the questionnaire regarding the questions about working conditions, including “reliability on teammates if it is getting difficult at work” (73.4%, 918/1250), “sufficient amount of protective clothing including face masks” (67.9%, 849/1250) and “enough staff for the current workload” (52.7%, 659/1250); the participants strongly or quite strongly agreed with these statements. Of all the respondents, 40.2% (502/1250) strongly or quite strongly agreed that they were able to recover sufficiently during their leisure time.

In the questions about potential COVID-19 problems, 20.6% (254/1236) of participants reported being afraid of becoming infected with COVID-19 (level of agreement: strongly or quite strongly). The fear of infecting family members was reported twice as frequently (41.9%, 518/1236). Fear of work was agreed on by only 14.4% (178/1236) of participants. A large proportion of participants agreed strongly or quite strongly with the questions about “burden of change of work tasks” (43.9%, 542/1236) and “burden of an increase in the quantity of work” (34.5%, 426/1236). The majority of participants (55.8%, 690/1236) also indicated that they felt protected by the measures taken by their employer (level of agreement: strongly or quite strongly). Only a relatively small proportion of participants reported increased alcohol (5.4%, 67/1236) and cigarette (4.4%, 54/1236) consumption.

The results of the multiple linear regression models are shown in Table 4. The substantial factors (significant (*p* < .05) and clinically relevant (β score ≥ .100) independent variables) for an increase in depressive symptoms (Model 1) and emotional exhaustion (Model 3) were the burden of an increase in the quantity of work and a fear of work. The fear of becoming infected also lead to increased symptoms of generalized anxiety disorder (Model 2). In contrast, an optimistic attitude, perceived emotional and social support, a higher sense of coherence, a higher quality of life, and sufficient relaxation in leisure time were associated with less of an increase in depressive symptoms, generalized anxiety disorder symptoms and emotional exhaustion.

**Table 4:** Multiple linear regression analysis of factors associated with depressive symptoms, generalized anxiety disorder symptoms and emotional exhaustion

Variables	Model 1: PHQ-2 (sum score) ***n*** = 1144 ***R***^***2***^_**adj**_ = .392, F = 31.67, ***p*** = <.001				Model 2: GAD-2 (sum score) ***n*** = 1140 ***R***^***2***^_**adj**_ = .390, F = 31.39, ***p*** = <.001				Model 3: MBI-EE (sum score) ***n*** = 1150 ***R***^***2***^_**adj**_ = .431, F = 37.26, ***p*** = <.001			
	B	SE	β	p	B	SE	β	p	B	SE	β	p
**Sociodemographic and occupational characteristics**												
Sex (male/Ref. female)	.167	.101	.041	.098	−.162	.109	−.037	.138	.570	.852	.016	.504
Age (≥31/Ref. ≤ 30)	−.116	.106	−.029	.274	.114	.114	.027	.316	−.755	.896	−.022	.400
Housing situation/living alone (yes/Ref. no)	−.074	.083	−.023	.376	−.112	.089	−.033	.209	.702	.700	.025	.316
Caring for chronically ill or handicapped family (yes/Ref. no)	−.055	.073	−.018	.457	.042	.079	.013	.596	−.287	.616	−.011	.641
Children (yes/Ref. no)	.017	.076	.007	.819	.193	.081	.069	**.018**	−.405	.637	−.018	.525
Immigrant background (no/Ref. yes)	.055	.097	.013	.573	−.001	.104	.000	.995	−.311	.816	−.009	.703
Risk group (yes/Ref. no)	−.018	.067	−.007	.789	.057	.071	.020	.423	.154	.560	.007	.783
Mode of employment (full-time/Ref. part-time)	.103	.065	.041	.110	.229	.069	.086	**.001**	1.315	.544	.060	**.016**
**Psychological resources**												
Social Support	−.066	.009	−.190	**<.001**	−.045	.010	−.119	**<.001**	−.402	.077	−.132	**<.001**
Sense of Coherence	−.097	.012	−.208	**<.001**	−.107	.013	−.214	**<.001**	−.562	.100	−.139	**<.001**
Optimism	−.147	.025	−.149	**<.001**	−.233	.027	−.220	**<.001**	−1.332	.207	−.154	**<.001**
Quality of Life	−.257	.048	−.151	**<.001**	−.243	.051	−.134	**<.001**	−1.996	.400	−.135	**<.001**
**Working conditions and potential problems in the COVID-19 pandemic**												
Sufficient amount of protective clothing including face masks	.045	.027	.045	.090	.018	.028	.017	.531	.508	.223	.058	**.023**
Enough staff for the current workload	.011	.022	.012	.631	.041	.024	.043	.089	−.346	.188	−.044	.066
Sufficient recreation during leisure time	−.082	.027	−.083	**.002**	−.099	.029	−.093	**.001**	−1.020	.228	−.118	**<.001**
Reliability on teammates if it is getting difficult at work	−.068	.029	−.059	**.021**	−.046	.031	−.038	.137	−.762	.246	−.076	**.002**
Fear of becoming infected	.015	.036	.014	.667	.138	.038	.120	**<.001**	.733	.301	.078	**.015**
Fear of infecting family	−.010	.030	−.010	.748	.019	.032	.020	.549	−.177	.252	−.022	.481
Fear of work	.127	.031	.121	**<.001**	.099	.033	.088	**.003**	1.210	.258	.132	**<.001**
Burden of change of work tasks	.086	.025	.093	**.001**	.057	.027	.058	**.035**	.660	.213	.082	**.002**
Burden of an increase in the quantity of work	.114	.027	.121	**<.001**	.099	.029	.098	**.001**	1.734	.224	.211	**<.001**
Feeling of being protected by employer	−.014	.030	−.013	.651	.003	.033	.002	.930	−.349	.256	−.037	.172
Increased cigarette consumption	.091	.041	.057	**.025**	.040	.043	.023	.355	−.222	.338	−.016	.512
Increased alcohol consumption	.070	.035	.051	**.048**	.069	.038	.047	.070	.222	.296	.019	.453

*Abbreviations*: *PHQ-2* Patient Health Questionnaire-2, *GAD-2* Generalized Anxiety Disorder, *MBI-EE* Maslach Burnout Inventory-Emotional Exhaustion subscale

Ref. = reference group

## Discussion

To the best of our knowledge, this is the first study to examine the impact of the COVID-19 pandemic on employees working with chronic pediatric patients in interdisciplinary teams. However, it should be noted that the results of studies conducted in the context of the pandemic must be compared with caution, as a variety of factors, such as the exact time of the survey, the method of recruitment, the study population and country-specific characteristics, must be taken into account. Comparability is also limited in our study because we did not study a purely medical field; our study took place at an interface between the educational and medical systems. Nevertheless, we believe it is important to consider this area, and not only at the staff in acute care clinics who are the most directly and obviously affected. Our analyses show that nearly half of the employees in pediatric development services felt burdened and emotionally exhausted by the pandemic; some even exhibited clinically relevant depressive and anxiety symptoms. Our investigated clientele has some special features. On the one hand, they are HCWs who often accompany the patients and their families for many years; therefore, they may also have a certain affinity for their patients. This may arise from close and frequent contact, as is the case with regular fostering in EICs, or from a corresponding high level of professional expertise at an SPC as a tertiary referral center. The staff are typically no longer concerned with the care of acutely ill patients, but rather precisely with chronic courses. Another special feature is interdisciplinary work in a multidisciplinary team. While in the usual clinical setting, even in acute medicine, several professional groups often work together, the interdisciplinary approach in social pediatrics differs from other fields [27]. In a recent study on interdisciplinarity (University of Aachen, RWTH, personal note), which will soon be submitted for publication, it was found that employees in this area felt they belonged to the entire team rather than just their own professional group. It is possible that this particular feature may also be reflected in the high values for the reliability of team members.

There are somewhat conflicting results in the literature concerning direct exposure to COVID-19 patients. Some authors have found a higher level of trauma in those directly exposed or at risk for exposure [28, 29], whereas others have found lower levels of trauma in frontline nurses than both the general public and non-frontline nurses [30]. Here, attention should always be paid to which mental construct was examined. There are several areas, such as stress, anxiety, exhaustion, etc., that should not be mixed but instead considered in a differentiated manner. Our survey showed a significant increase in burden in all occupational groups, although the increase did not always meet our expectations at first glance. For example, we found a relatively small increase among psychologists and physicians compared to other occupational groups. In part, this can be explained by the fact that physicians already reported a higher baseline burden before the pandemic; thus, the relative increase was smaller [31]. Speculatively, other explanatory models would also be conceivable. For example, physicians are confronted with acute crises or catastrophic situations more frequently than other occupational groups during their training, which typically takes place in clinics in Germany, and are then better adapted to handle such scenarios in a repeat case. In addition, a better integration of the multitude of medical information could contribute to better coping and thus reduced stress compared to other occupational groups. Psychologists, on the other hand, might already have better knowledge of their own resources and mindfulness through their studies and thus have access to more resources for dealing with stressful situations or scenarios. However, from our point of view, one of the most important findings is that burden can affect all occupational groups, even those for whom it is not necessarily expected in the first place; this can be the case to a considerable extent.

Now, what might account for the burden among employees who are not directly confronted with acutely ill patients? The employees may show a certain identification with the patients and families they have accompanied for years and now observe (speculatively) that their patients are “falling into a hole” and are no longer able to get any support, but they are also not allowed to provide this themselves because the facility may be closed and official restrictions applied. An indication in this direction could also be that the employees surveyed were more concerned about others than about themselves: while 20.6% reported at least some fear of infecting themselves, the rate for fear of infecting family members was 41.9%.

If we consider the anxiety symptom scores of our study, they were in the elevated range compared to the German norming sample [18]. Increased anxiety was also evident in other studies, although anxiety did not always correlate with direct experience or actual increased risk of exposure [32]. Thus, other authors have also observed psychological and social influencing factors, which in turn enable interventions [32]. Similar results were also reported from initial research in China regarding emotional exhaustion as measured by the MBI. Here too, the employees who directly treated the infected patients showed lower values (i.e., were less exhausted) than those in the “normal wards” [33]. A high level of strain in work areas that were not directly involved with acutely ill patients, and in which there was sometimes no patient contact at all, was also reflected in the results of the PHQ-2 and the MBI-EE. If we now compare our results with other studies, the picture is not entirely consistent, which, as already mentioned, may also be due to differences in conditions (e.g., timing of the survey in the course of the pandemic, recruitment, gender distribution, etc.). A survey that was open to the entire population in Germany showed even higher scores on the PHQ-2 in March 2020—an earlier phase of the lockdown in Germany—with significantly more responses and higher scores from women than men [32]. The higher scores reported in this study could also be due to the open nature of the survey: a survey that is open to all may be more likely to attract people with higher stress levels than those with fewer problems. However, this idea must remain somewhat speculative, since, on the other hand, those with very high workloads and the resulting stress simply may not have time to participate in surveys. We assume that it is not the absolute values that are decisive, but rather the comparison to the original reference population and the consideration of the changes in these values ​​over the course of the different stages of the pandemic.

When looking at the regression model, further possible approaches for (future) prevention emerge. With many employees virtually unable to work, the workload results are likely to reflect an uneven distribution of work in this situation. In the future, distribution and responsibility plans should provide relief on the one hand and a better workload on the other. This better workload—“I am needed”—might also increase the sense of coherence. At the political or corporate level in particular, further measures should be taken to provide emotional and social support, as this emerged as one of the key resilience factors in our study. Another possibility would be better networking of respective employees, also across institutions, whereby new media can certainly contribute in the age of physical distancing.

One possible conclusion from this is that not only are targeted interventions useful and should be offered, but it may well make sense to adopt general preventive approaches for all. A very important resource in our work context also seems to be the reliability of the team members. Although there are few good, controlled studies that have examined interventions specifically in an epidemic or pandemic event [34], it is possible to draw on analogous interventions that have proven effective in other crisis situations. There are some programs that provide mental health support and are also well evaluated when it comes to burnout or emotional exhaustion, for example [35]. When such interventions have been implemented and offered, they have also proven helpful in the context of the pandemic [36]. Some of these services can also be implemented using digital options, which is especially important during a pandemic.

### Limitations

Because of the recruitment method used for participants in our survey, we can say little about whether our results are representative of the entire workforce in the field. A bias is conceivable in both directions. On the one hand, clearly stressed employees may have participated to a greater extent in order to make their opinions known and to articulate their needs. On the other hand, it is equally conceivable that these employees could not have been reached in the first place due to illness or withdrawal. Due to the heterogeneity of the group studied, there is also no representative data, e.g., from employers. However, when we consider the data from the survey of SPCs, at least the demographics of our participants, including the distribution of their occupational groups, fit well with the overall workforce in SPCs [37]. The cross-sectional design also implies a limitation as far as a causal relationship is concerned. Therefore, a longitudinal follow-up will also be performed to better assess these issues.

## Conclusion

Most previously published studies have focused on the situation of employees who are on the immediate front lines of the pandemic. We were able to show with our data that employees who were not directly involved in the care of acutely ill patients also bore a high burden in the context of the COVID-19 pandemic. In our view, however, it cannot be a question of who is now the most burdened, but rather of what methods can be used to counteract these burdens. We consider general prevention approaches to be appropriate.

## Data Availability

The datasets generated during and/or analyzed during the current study are available from the corresponding author on reasonable request.

## Abbreviations

Aks: Arbeitskreis für Vorsorge- und Sozialmedizin/the working group for preventive and social medicine
BMBF: Bundesministerium für Bildung und Forschung/Federal Ministry of Education and Research
COVID-19: Coronavirus disease 2019
EIC: Early intervention center
ESSI-D: ENRICHD Social Support Inventory-Deutsch
GAD-2: Generalized Anxiety Disorder-2
HCW: Healthcare worker
LMIC: Low- and middle-income country
MBI-EE: Maslach Burnout Inventory-Emotional Exhaustion subscale
MBI-HSS: Maslach Burnout Inventory-Human Services Survey
NUM: Network University Medicine
SOP-2: Scale Optimism-Pessimism-2
PHQ-2: Patient Health Questionnaire-2
RWTH: Rheinisch-Westfälische Technische Hochschule Aachen
SD: Standard deviation
SOC-3: Sense of Coherence Scale-3
SPC: Social Pediatric Center
WHOQOL-BREF: World Health Organization Quality of Life
WMA: World Medical Association

## References

[1] Shreffler J, Petrey J, Huecker M (2020). The impact of COVID-19 on healthcare worker wellness: a scoping review. West J Emerg Med.

[2] Wanigasooriya K, Palimar P, Naumann DN, Ismail K, Fellows JL, Logan P, Thompson CV, Bermingham H, Beggs AD, Ismail T (2020). Mental health symptoms in a cohort of hospital healthcare workers following the first peak of the COVID-19 pandemic in the UK. BJPsych Open.

[3] Kotabagi P, Fortune L, Essien S, Nauta M, Yoong W (2020). Anxiety and depression levels among pregnant women with COVID-19. Acta Obstet Gynecol Scand.

[4] Niedzwiedz CL, Green MJ, Benzeval M, Campbell D, Craig P, Demou E, Leyland A, Pearce A, Thomson R, Whitley E, Katikireddi SV (2021). Mental health and health behaviours before and during the initial phase of the COVID-19 lockdown: longitudinal analyses of the UK household longitudinal study. J Epidemiol Community Health.

[5] Koopmann A, Ekaterini G, Falk K, Hillemacher T (2020). Did the general population in Germany drink more alcohol during the COVID-19 pandemic lockdown?. Alcohol Alcohol.

[6] Barello S, Caruso R, Palamenghi L, Nania T, Dellafiore F, Bonetti L, Silenzi A, Marotta C, Graffigna G (2021). Factors associated with emotional exhaustion in healthcare professionals involved in the COVID-19 pandemic: an application of the job demands-resources model. Int Arch Occup Environ Health.

[7] Moitra M, Rahman M, Collins PY, Gohar F, Weaver M, Kinuthia J, Rössler W, Petersen S, Unutzer J, Saxena S, Huang KY, Lai J, Kumar M (2021). Mental health consequences for healthcare workers during the COVID-19 pandemic: a scoping review to draw lessons for LMICs. Front Psychiatry.

[8] Jang Y, You M, Lee H, Lee M, Lee Y, Han JO, Oh JH (2021). Burnout and peritraumatic distress of healthcare workers in the COVID-19 pandemic. BMC Public Health.

[9] Salvagioni DAJ, Melanda FN, Mesas AE, González AD, Gabani FL, Andrade SM (2017). Physical, psychological and occupational consequences of job burnout: a systematic review of prospective studies. PLoS One.

[10] Kim JS, Choi JS (2016). Factors influencing emergency Nurses' burnout during an outbreak of Middle East respiratory syndrome coronavirus in Korea. Asian Nurs Res (Korean Soc Nurs Sci).

[11] Ball J, Nehme Z, Bernard S, Stub D, Stephenson M, Smitha K (2020). Collateral damage: hidden impact of the COVID-19 pandemic on the out-of-hospital cardiac arrest system-of-care. Resuscitation.

[12] Saso A, Skirrow H, Kampmann B (2020). Impact of COVID-19 on immunization Services for Maternal and Infant Vaccines: results of a survey conducted by imprint-the Immunising pregnant women and infants network. Vaccines.

[13] Santarone K, McKenney M, Elkbuli A (2020). Preserving mental health and resilience in frontline healthcare workers during COVID-19. Am J Emerg Med.

[14] Deutsche Gesellschaft für Sozialpädiatrie und Jugendmedizin (DGSPJ): Sozialpädiatrische Zentren. https://www.dgspj.de/institution/sozialpaediatrische-zentren/. Accessed 11 Nov 2021.

[15] Vereinigung für interdisziplinäre Frühförderung – Bundesvereinigung (VIFF). www.viff-fruehfoerderung.de. Accessed 11 Nov 2021.

[16] aks Kinderdienste. https://www.aks.or.at/fachleute/kinder-und-jugendpsychiatrie/. Accessed 11 Nov 2021.

[17] Morawa E, Schug C, Geiser F, Beschoner P, Jerg-Bretzke L, Albus C, Weidner C, Hiebel N, Borho A, Erim Y (2021). Psychosocial burden and working conditions during the COVID-19 pandemic in Germany: the VOICE survey among 3678 health care workers in hospitals. J Psychosom Res.

[18] Löwe B, Wahl I, Rose M, Spitzer C, Glaesmer H, Wingenfeld K, Schneider A, Brähler EA (2010). A 4-item measure of depression and anxiety: validation and standardization of the patient health Questionnaire-4 (PHQ-4) in the general population. J Affect Disord.

[19] Maslach C, Jackson SE (1981). The measurement of experienced burnout. J Occup Behav.

[20] Büssing A, Perrar KM (1992). Die Messung von Burnout. Untersuchung einer deutschen Fassung des Maslach Burnout Inventory (MBI-D). Diagnostica.

[21] Maslach C, Jackson SE, Leiter MP (1996). Maslach burnout inventory manual.

[22] Kendel F, Spaderna H, Sieverding M, Dunkel A, Lehmkuhl E, Hetzer R, Regitz-Zagrosek V (2011). Eine deutsche Adaptation des ENRICHD Social Support Inventory (ESSI) Teststatistische Überprüfung an kardialen Patienten. Diagnostica.

[23] Schmalbach B, Tibubos AN, Zenger M, Hinz A, Brähler E (2020). Psychometrische evaluation und Normwerte einer Ultrakurzform der sense of coherence scale “SOC-3”. Psychother Psychosom Med Psychol.

[24] Angermeyer C, Kilian K, Matschinger H (2002). Deutschsprachige Version der WHO Instrumente zur Erfassung von Lebensqualität WHOQOL-100 und WHOQOL-BREFM. Z Med Psychol.

[25] Kemper CJ, Beierlein C, Kovaleva A, Rammstedt B (2013). Entwicklung und Validierung einer ultrakurzen Operationalisierung des Konstrukts Optimismus-Pessimismus – Die Skala Optimismus-Pessimismus-2 (SOP2). Diagnostica.

[26] Cohen J (1988). Statistical power analysis for the behavioral sciences.

[27] Hoffmann A, Floß S (2020). Interdisziplinarität im SPZ – unser Markenzeichen. Kinderärztliche Praxis.

[28] Guo J, Liao L, Wang B, Li X, Guo L, Tong Z, Guan Q, Zhou M, Wu Y, Zhang J, Dardik A, Gu Y (2021). Psychological effects of COVID-19 on hospital staff: a national cross-sectional survey in mainland China. Vasc Invest Ther.

[29] Liu Z, Han B, Jiang R, Huang Y, Ma C, Wen J, et al. Mental health status of doctors and nurses during COVID-19 epidemic in China. SSRN. 2020. 10.2139/ssrn.3551329.

[30] Li Z, Ge J, Yang M, Feng J, Qiao M, Jiang R, Bi J, Zhan G, Xu X, Wang L, Zhou Q, Zhou C, Pan Y, Liu S, Zhang H, Yang J, Zhu B, Hu Y, Hashimoto K, Jia Y, Wang H, Wang R, Liu C, Yang C (2020). Vicarious traumatization in the general public, members, and non-members of medical teams aiding in COVID-19 control. Brain Behav Immun.

[31] Patel RS, Bachu R, Adikey A, Malik M, Shah M (2018). Factors related to physician burnout and its consequences: a review. Behav Sci.

[32] Petzold MB, Bendau A, Plag J, Pyrkoschet L, Maricic LM, Betzler F, Rogoll J, Große J, Ströhle A (2020). Risk, resilience, psychological distress, and anxiety at the beginning of the COVID-19 pandemic in Germany. Brain Behav.

[33] Wu Y, Wang J, Luo C, Hu S, Lin X, Anderson AE, Bruera E, Yang X, Wie S, Qian YA (2020). A comparison of burnout frequency among oncology physicians and nurses working on the frontline and usual wards during the COVID-19 epidemic in Wuhan, China. J Pain Symptom Manag.

[34] Pollock A, Campbell P, Cheyne J, Cowie J, Davis B, McCallum J, McGill K, Elders A, Hagen S, McClurg D, Torrens C, Maxwell M (2020). Interventions to support the resilience and mental health of frontline health and social care professionals during and after a disease outbreak, epidemic or pandemic: a mixed methods systematic review. Cochrane Database Syst Rev.

[35] West CP, Dyrbye LN, Erwin PJ, Shanafelt TD (2016). Interventions to prevent and reduce physician burnout: a systematic review and meta-analysis. Lancet.

[36] Mellins CA, Mayer LES, Glasofer DR, Devlin MJ, Albano AM, Nash SS, Engle E, Cullen C, Ng WYK, Allmann AE, Fitelson EM, Vieira A, Remien RH, Malone P, Wainberg ML, Baptista-Neto L (2020). Supporting the well-being of health care providers during the COVID-19 pandemic: the CopeColumbia response. Gen Hosp Psychiatry.

[37] Kretzschmar C (2020). Sind SPZ eine Blackbox?. Kinderärztliche Praxis.

